# Damaged DNA Binding Protein 2 Plays a Role in Breast Cancer Cell Growth

**DOI:** 10.1371/journal.pone.0002002

**Published:** 2008-04-23

**Authors:** Zilal Kattan, Sophie Marchal, Emilie Brunner, Carole Ramacci, Agnès Leroux, Jean Louis Merlin, Lionel Domenjoud, Michel Dauça, Philippe Becuwe

**Affiliations:** 1 Laboratoire de Biologie Cellulaire du Développement, EA 3446 Université Henri Poincaré-Nancy Université, Vandoeuvre-lès-Nancy, France; 2 Centre Alexis Vautrin, UMR 7039 Institut Polytechnique de Lorraine/Université Henri Poincaré-Nancy Université/ CNRS, Vandoeuvre les Nancy, France; 3 Unité de Biologie des Tumeurs du Centre Alexis Vautrin, EA3452 Nancy Université, Vandoeuvre lès Nancy, France; Ordway Research Institute, United States of America

## Abstract

The Damaged DNA binding protein 2 (DDB2), is involved in nucleotide excision repair as well as in other biological processes in normal cells, including transcription and cell cycle regulation. Loss of DDB2 function may be related to tumor susceptibility. However, hypothesis of this study was that DDB2 could play a role in breast cancer cell growth, resulting in its well known interaction with the proliferative marker E2F1 in breast neoplasia. DDB2 gene was overexpressed in estrogen receptor (ER)-positive (MCF-7 and T47D), but not in ER-negative breast cancer (MDA-MB231 and SKBR3) or normal mammary epithelial cell lines. In addition, DDB2 expression was significantly (3.0-fold) higher in ER-positive than in ER-negative tumor samples (*P = *0.0208) from 16 patients with breast carcinoma. Knockdown of DDB2 by small interfering RNA in MCF-7 cells caused a decrease in cancer cell growth and colony formation. Inversely, introduction of the DDB2 gene into MDA-MB231 cells stimulated growth and colony formation. Cell cycle distribution and 5 Bromodeoxyuridine incorporation by flow cytometry analysis showed that the growth-inhibiting effect of DDB2 knockdown was the consequence of a delayed G1/S transition and a slowed progression through the S phase of MCF-7 cells. These results were supported by a strong decrease in the expression of S phase markers (Proliferating Cell Nuclear Antigen, cyclin E and dihydrofolate reductase). These findings demonstrate for the first time that DDB2 can play a role as oncogene and may become a promising candidate as a predictive marker in breast cancer.

## Introduction

Damaged DNA Binding protein 2 (DDB2) is a 48-kDa protein originally identified as a component of the damage-specific DNA-binding heterodimeric complex DDB, which is involved in nucleotide excision repair of UV-induced DNA damage through interaction with DDB1 (127- kDA protein) or CSA proteins [1]. It works also in association with other proteins of the repair system, including the XPC-hHR23B heterodimer, XPA and replication protein A [2], [3]. DDB2 shares homology with chromatin reorganizing proteins [4] and interacts with the CBP/p300 histone acetyl transferases and STAGA complex [5], [6], consistent with a function in the remodelling of chromatin to allow efficient repair in the vicinity of DNA lesions. In addition, DDB2 participates in global NER by recruiting ubiquitinating enzymes, such as the E3 ubiquitin ligase cullin 4A [7].

Human DDB2 is involved in other cellular processes, including transcription and cell cycle regulation. It has been demonstrated that DDB2 acts as a co-factor of the transcription factor E2F1 [8] and that it is associated with the transcriptional coactivator protein CBP/p300 and the chromatin-acetylating transcription coactivator STAGA complex [5], [6]. DDB2 is a downstream target of BRCA1 and p53, which regulate its gene expression [9]–[11], suggesting that DDB2 could also be involved in cell cycle regulation. DDB2 is a cell cycle-regulated protein in normal cells since it is undetectable in nondividing cells but its level increases in the mid-G1 phase and peaks at the G1/S boundary, before dropping significantly in the S phase [12]. The cell cycle-regulation of DDB2 levels involves ubiquitin-proteasome pathway-mediated proteolysis, through the interaction between DDB2 and cullin 4A [7], [12], [13].

Loss of DDB2 function in normal cells is related to tumor development susceptibility. Mutations in the DDB2 gene leading to its loss of function are responsible for the phenotypic features of xeroderma pigmentosum group E (XP-E) patients, characterized by malignant skin tumors [14]-[16]. In addition, DDB2-deficient mice not only were hypersensitive to UV-induced skin carcinogenesis but also developed a high rate of broad spectrum spontaneous malignant tumors in internal organs, in the absence of UV irradiation or added carcinogen. These observations suggested that DDB2 may play a role as a downstream mediator in the tumor suppression pathways of p53 and BRCA1 [17], [18]. This suggests a role of DDB2 as a tumor suppressor in normal cells, through protecting against cancer by regulating the cell cycle and by increasing apoptosis rather than by direct participation in the repair of DNA damage [19].

Even if DDB2 is considered as a tumor suppressor, we proposed that this protein could play a role in breast cancer. This hypothesis was based on a previous study showing an interaction between DDB2 and the transcription factor E2F1, a proliferative marker in breast carcinoma [20]. Also, the aim of this study was to examine the expression of the DDB2 gene in various breast cancer cell lines and in tumors from patients. The surprising results showing an overexpression of DDB2 in ER-positive but not in ER-negative breast cancers led us to investigate the role of this protein in breast cancer cells. This study provides evidence for the first time that DDB2 plays an important role in cell cycle progression of breast cancer cells.

## Results

### DDB2 gene is overexpressed in ER-positive breast cancer cell lines

**Figure 1:**
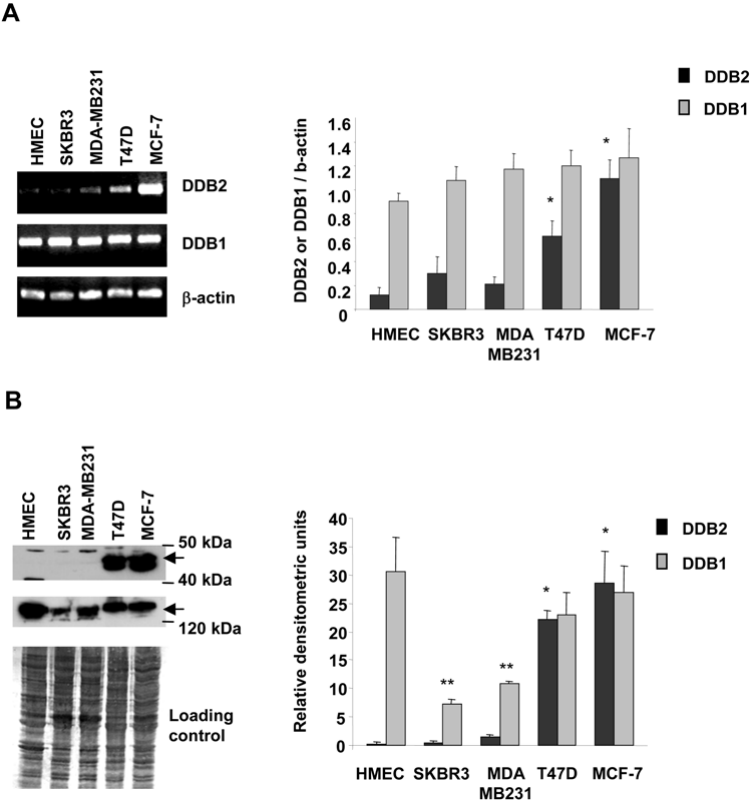
DDB2 is overexpressed in human ER-positive breast cancer cell lines. (A) Total RNA was extracted from cells, then subjected to semiquantitative RT-PCR analysis. The relative levels of DDB1 and DDB2 mRNAs were normalized to those of β-actin mRNA. (B) DDB1 and DDB2 proteins were analysed in the total protein (50 µg) extracted from cells by Western blotting, using polyclonal anti-DDB1 and anti-DDB2 antibodies. Coomassie blue membrane staining was used as the protein loading control. Results are representative of three independent experiments. Relative band intensities for RT-PCR and Western blot analysis were quantified by densitometry. Data from RT-PCR analysis are expressed as the ratio for DDB1 or DDB2 mRNA levels/ β-actin mRNA levels. Data from Western blot analysis are expressed as relative densitometric units. Statistically significant differences from the HMEC values for DDB1 and DDB2 are indicated as ** and *P<0.05, respectively.

The expression of DDB2 was assessed in both the ER-negative (MDA-MB231 and SKBR3) and ER-positive (MCF-7 and T47D) breast cancer cells and was compared to that in the normal human mammary epithelial cells (HMEC), at both the transcriptional and the translational levels (Figure 1). The DDB2 mRNA level estimated by RT-PCR analysis was 9.1-fold higher for MCF-7 and 5.1-fold higher for T47D cell lines than for HMEC cells (Figure 1A). No significant difference was observed between the DDB2 mRNA levels of the ER-negative (MDA-MB231 and SKBR3) cancer cells and the HMEC cells. These results correlated with the DDB2 protein content. Western blot analysis showed that the MCF-7 and T47D cells had similar and very high levels of DDB2 protein, in contrast to the very low levels in MDA-MB231, SKBR3 and HMEC cells (Figure 1B). No significant difference was observed between the DDB1 mRNA levels of the cell lines studied. However, the MCF-7 cells had respectively 3.7- and 2.5-fold higher levels of DDB1 protein than the SKBR3 and MDA-MB231 cells. No significant difference was observed between the DDB1 protein levels of the MCF-7, T47D and HMEC cell lines. The MCF-7 cells expressing the highest basal DDB2 levels were submitted to in situ immunofluorescence in order to analyse the subcellular localization of DDB1 and DDB2. Whereas DDB1 was localized in both the nucleus and the cytoplasm, DDB2 was exclusively found in the nucleus (see Supplemental Figure S1).

### DDB2 gene is expressed in human breast tumors

**Figure 2:**
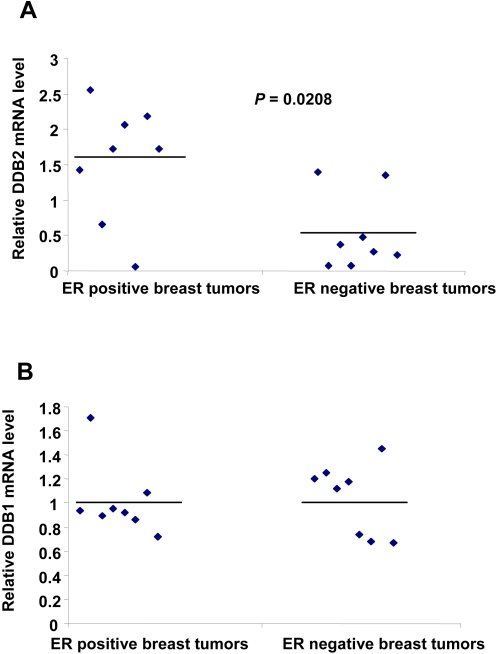
DDB2 is expressed in human breast tumors from patients. Total RNA was extracted from eight ER-positive and eight ER-negative breast cancer samples, then subjected to semiquantitative RT-PCR analysis. The relative levels of DDB1 and DDB2 mRNAs were normalized to those of β-actin mRNA. Statistically significant differences between ER-positive and ER-negative samples are indicated as *P<*0.05. The mean values are indicated by a bar in the graph for each group of tumors and DDB1 or DDB2 mRNA levels.

As DDB2 appeared to be detected strongly in ER-positive breast cancer cell lines, we next examined the DDB2 mRNA level in breast cancer samples from 16 patients (eight positive and eight negative for ER expression) using semiquantitative RT-PCR. In concordance with the results from breast cancer cell lines, the relative DDB2 mRNA level was significantly 3.0-fold higher in ER-positive than in ER-negative breast tumor samples (P = 0.0208). The mean value of relative DDB2 mRNA level was 1.55±0.82 for ER-positive breast tumors as compared to 0.53±0.54 for ER-negative breast tumors. Five of the eight ER-positive samples showed a higher relative DDB2 mRNA level than the mean value, whereas one sample did not express DDB2 (Figure 2).

### DDB2 knockdown leads to a decrease in the growth of ER-positive breast cancer cells

**Figure 3:**
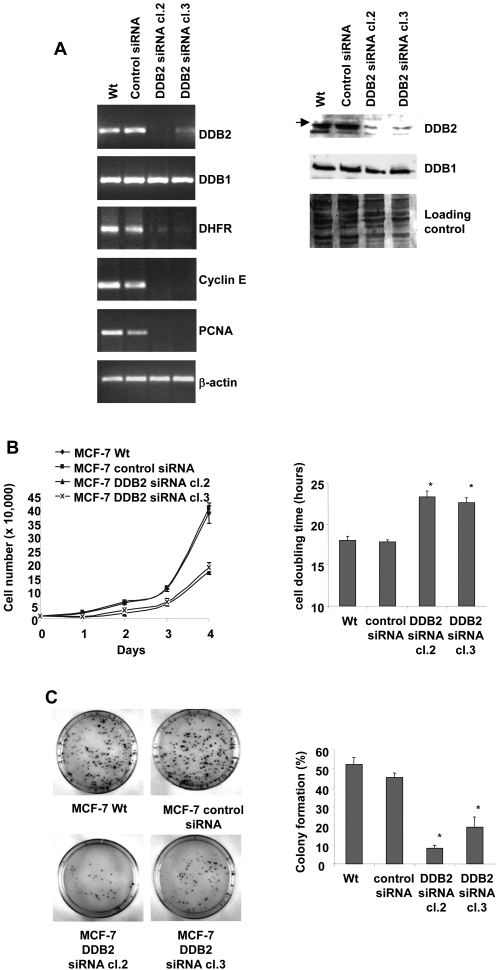
DDB2 knockdown affects the growth and colony formation of MCF-7 cells. (A) Generation of MCF-7 cell clones stably expressing DDB2 siRNA. Total RNA was extracted from parental MCF-7 cells (Wt) and from cells stably transfected with either the DDB2-siRNA vector (clones cl.2 and cl.3) or the scrambled siRNA vector (control siRNA), then subjected to RT-PCR analysis. The relative levels of DDB1, DDB2, DHFR, cyclin E and PCNA mRNAs were normalized to those of β-actin mRNA. DDB1 and DDB2 protein levels were analysed in the total protein (50 µg) extracted from MCF-7 cell clones stably expressing DDB2 siRNA and from control cells, by Western blotting using polyclonal anti-DDB1 and anti-DDB2 antibodies. Coomassie blue membrane staining was used as the protein loading control for Western blot analysis. (B) Parent cells (Wt), control siRNA-transfected MCF-7 cells and the two DDB2 siRNA-transfected cell clones (DDB2 siRNA cl.2 and cl.3) were plated in 24-well dishes (1×10^4^ per well) in complete medium and cell numbers were counted for 4 days. Means are shown for three experiments. Cell population doubling time was calculated from the cell growth curve during the exponential growth phase. (C) Wt cells, control siRNA and DDB2 siRNA transfected cells were seeded (500 cells) in 100-mm dishes and grown for 12 days. Colonies with more than 50 cells were counted and data from three independent experiments were expressed as the % of colony formation = (colonies formed/cells seeded)×100%. Statistically significant differences from the parental (Wt) cell value are indicated as * *P*<0.05.

To understand the role of DDB2 in breast cancer cell growth, we applied RNA interference technology to knock down the overexpression of DDB2 in the MCF-7 cells. Three different duplexes of siRNA, targeting various sequences of DDB2 mRNA, were transfected individually into the MCF-7 cells at 100 nM for 24 h and the DDB2 protein level was analyzed by immunoblotting. All the DDB2 siRNA duplexes were able to knock down the expression of DDB2, albeit to varying degrees. This efficiency of these siRNA duplexes in the depletion of DDB2 expression was confirmed in COS-7 cells expressing stably Myc-His tagged DDB2 (see Supplemental Figure S2). The siRNA duplex 3, which exhibited the highest efficiency (about 90%) to deplete cellular DDB2 protein, was introduced into the RNAi-ready pSIREN vector for stable DDB2 suppression in the MCF-7 cells. Two stably DDB2 siRNA-transfected MCF-7 cell clones (cl.2 and cl.3) were isolated, where the DDB2 expression was strongly suppressed in contrast to that in the parent cells (Wt) and in the stably transfected cells with a scrambled siRNA duplex sequence (control siRNA) which were used as controls (Figure 3A and B). The expression of DDB1 was not affected in the transfected cells, in contrast to that in the Wt MCF-7 cells. Growth curves were assessed by seeding cells in 24-well tissue culture plates at 1×10^4^ cells/well and then cells were counted daily over a 4-day period. The two DDB2 deficient MCF-7 cell clones 2 and 3 showed an approximately similar growth rate and grew significantly more slowly than the Wt and the control siRNA-transfected MCF-7 cells. At day 4 after plating, the clones 2 and 3 exhibited respectively approximately 2.3- and 2.1-fold fewer cells than the control siRNA-transfected MCF-7 cells (Figure 3C). No significant difference was observed between the Wt and control siRNA-transfected MCF-7 cells. Cell population doubling times were calculated from the cell growth curves (Figure 3D). In accordance with the growth rates, the doubling times of the DDB2 deficient MCF-7 cell clones 2 and 3 (23.3 and 22.6 h, respectively) were longer than those observed for the Wt and control siRNA-transfected MCF-7 cells (18.0 and 17.8 h, respectively). As shown in Figure 3E and F, the decreases in cell growth rates were accompanied by a strong decrease in colony formation for the DDB2 deficient MCF-7 cell clones 2 and 3 (8.4 and 19.4%, respectively) compared to those of the Wt and control siRNA-transfected MCF-7 cells (52.4 and 45.5%, respectively). The decreases in cell growth rate and colony formation were associated with a decrease in the level of mRNA encoding specific S-phase markers, such as DHFR, cyclin E and PCNA, in the DDB2-deficient MCF-7 cell clones 2 and 3 (Figure 3A).

### DDB2 overexpression increases the growth of ER-negative breast cancer cells

**Figure 4:**
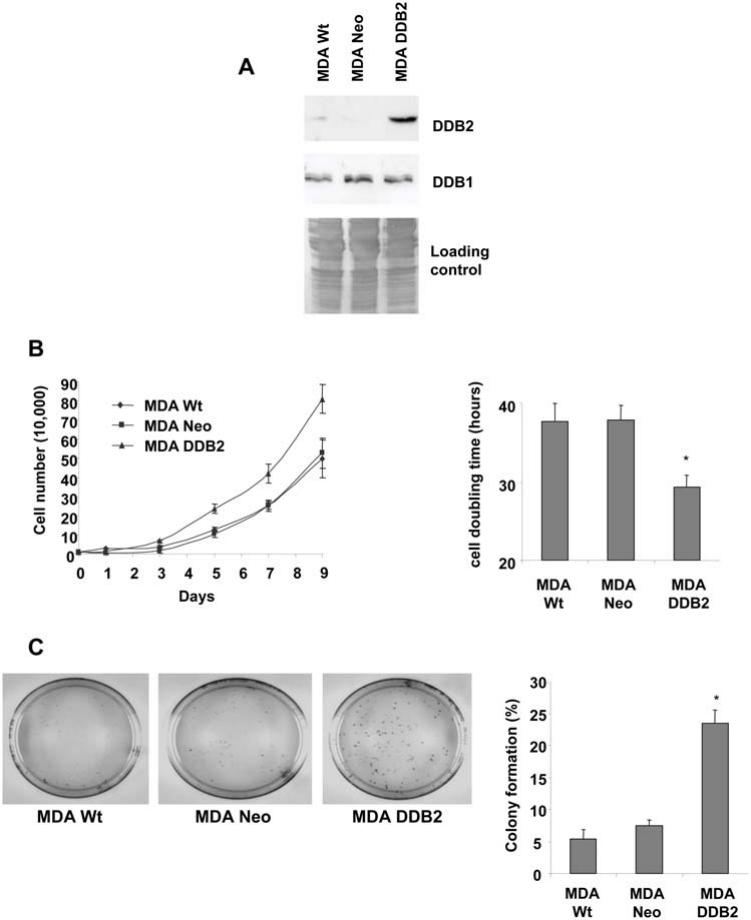
DDB2 overexpression increases the growth and colony formation of MDA-MB231 cells. (A) MDA-MB231 cells were stably transfected with the pcDNA 3(+) expression vector containing either DDB2 cDNA (MDA DDB2) or no insert (MDA Neo). The DDB2 protein level was assessed by Western blot analysis, using equal amounts of protein (50 µg) extracted from parent cells (MDA Wt), empty vector-transfected cells (MDA Neo) and DDB2-overexpressing cells (MDA DDB2), and using DDB2 polyclonal antibody. Coomassie blue membrane staining was used as the protein loading control. (B) Parent cells (MDA Wt), empty vector-transfected cells (MDA Neo) and DDB2-transfected cell clone (MDA DDB2) were plated in 24-well dishes (1×10^4^ per well) and cultured in complete medium. Cell numbers were counted on days 1, 3, 5, 7 and 9. Means are shown for three experiments. Cell population doubling times were calculated from the cell growth curves during the exponential growth phase. (C) MDA Wt, MDA Neo and MDA DDB2 cells were seeded (500 cells) in 100-mm dishes and grown for 12 days. Colonies with more than 50 cells were counted and data from three independent experiments were expressed as the % of colony formation = (colonies formed/cells seeded)×100%. Statistically significant differences from the MDA Wt cell value are indicated as * *P*<0.05.

The involvement of DDB2 in breast cancer cell growth was confirmed by introduction of the DDB2 gene into MDA-MB231 cells. These cells (MDA DDB2), control parent (MDA Wt) and empty vector-transfected cells (MDA Neo) were seeded in 24-well tissue culture plates at 1×10^4^ cells/well. The growth curve of the isolated MDA-MB231 cells overexpressing DDB2 (MDA DDB2) (Figure 4A) was assessed and compared to those of both control cell lines (MDA Wt and MDA Neo). The cells were then counted daily over a 9-day period. The MDA DDB2 cells grew faster, with approximately 1.7-fold more cells than the control Wt and Neo cell lines by day 9 after plating (Figure 4B). No significant difference was observed between the Wt and Neo control cells. In consequence, the cell population doubling time of the MDA DDB2 cells (29.4 h) was shorter (Figure 4C) than those observed for the MDA Wt and MDA Neo (37.7 and 37.9 h, respectively). In addition, colony formation was stimulated in the MDA DDB2 cells (23.5%) compared to that in the Wt and Neo control cells (5.3 and 7.5%, respectively) (Figure 4D and E).

### DDB2 knockdown promotes a decline in cell cycle progression at the G1/S transition in ER-positive breast cancer cells

**Figure 5:**
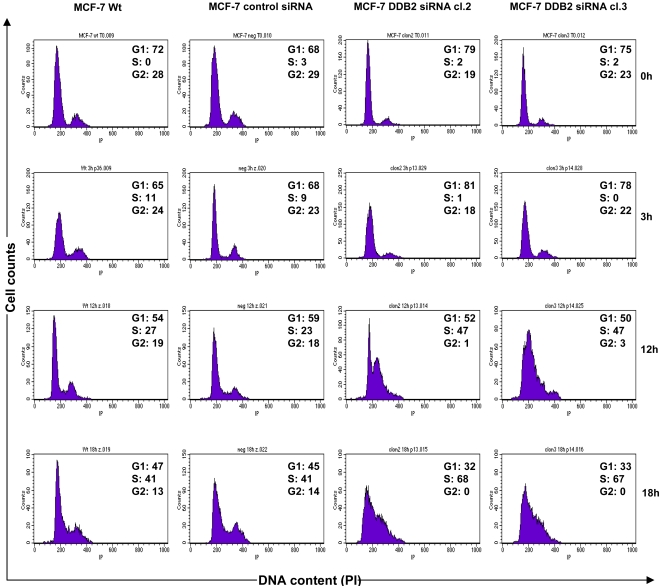
DDB2 knockdown affects cell cycle distribution of MCF-7 cells. Parent cells (Wt), control siRNA-transfected MCF-7 cells and the two DDB2 siRNA-transfected cell clones (DDB2 siRNA cl.2 and cl.3) were synchronized by serum starvation for 48h, and induced to re-enter the cell cycle by the addition of serum for 0, 3, 12 or 18h. MCF-7 cells were harvested for propidium iodide staining and analysed by FACS to determine the cell cycle fraction. FACS plots and data are representative of at least three separate experiments.

**Figure 6:**
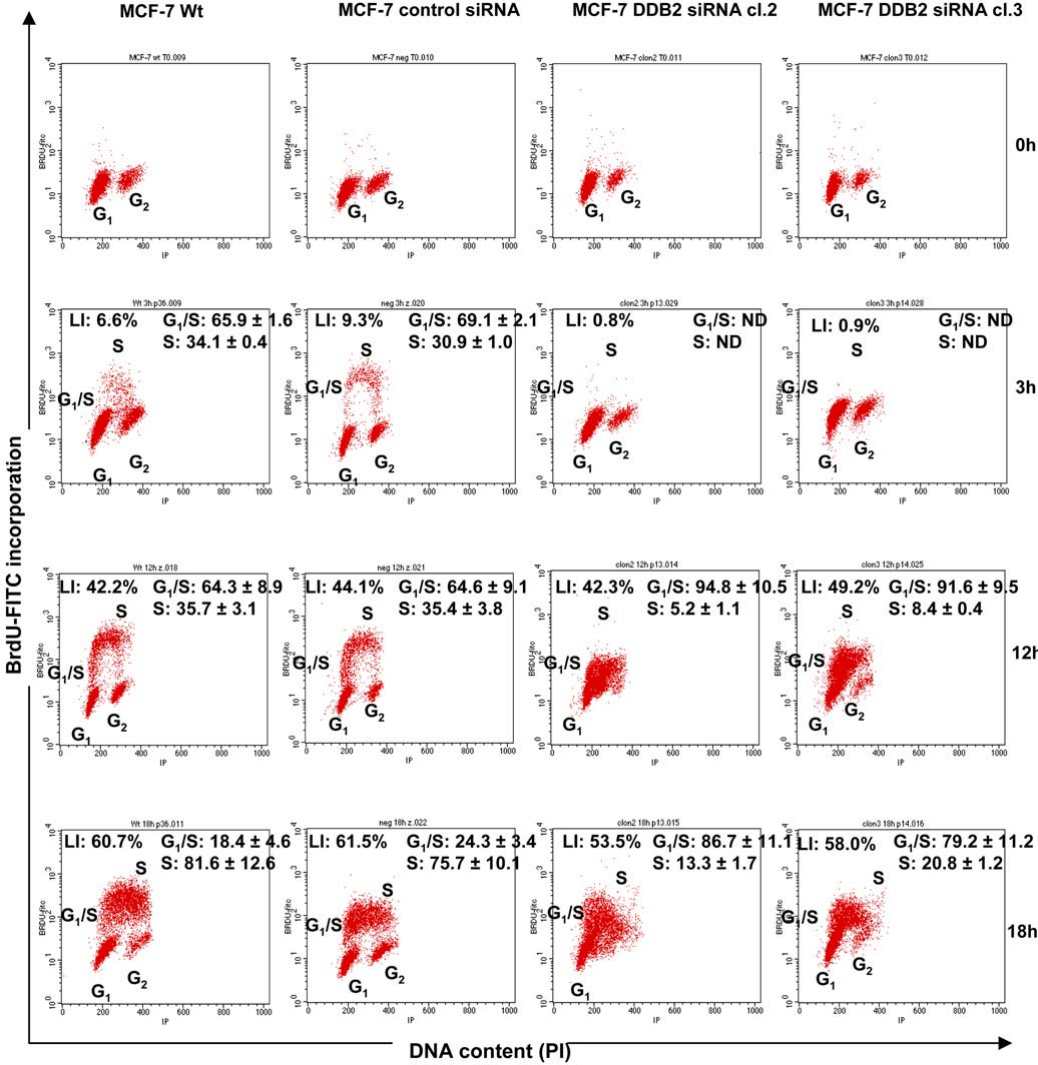
DDB2 knockdown decreases the ability of MCF-7 cells to re-enter the S phase of the cell cycle. Parent cells (Wt), control siRNA-transfected MCF-7 cells and the two DDB2 siRNA-transfected cell clones (DDB2 siRNA cl.2 and cl.3) were synchronized by serum starvation for 48h, and induced to re-enter cell cycle by the addition of serum for 0, 3, 12 or 18h. At the end of each of these periods after serum addition, MCF-7 cells were exposed to 5 BrdU for 20 min and were harvested for propidium iodide staining before being analysed by FACS for determination of the G1/S subpopulation and the S-phase fraction. FACS plots and data are representative of at least three separate experiments.

**Figure 7:**
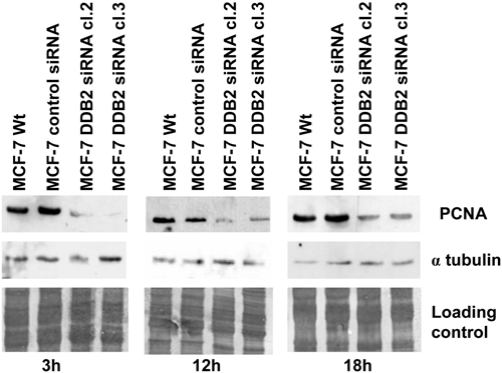
Analysis of PCNA expression in DDB2-deficient MCF-7 cells. Parent cells (Wt), control siRNA-transfected MCF-7 cells and the two DDB2 siRNA-transfected cell clones (DDB2 siRNA cl.2 and cl.3) were synchronized by serum starvation for 48h, and induced to re-enter the cell cycle by the addition of serum for 3, 12 or 18h. PCNA protein levels were analysed in the total protein (50 µg) extracted from different cells by Western blotting, using specific polyclonal antibodies. Membranes were then probed with specific polyclonal antibodies against tubulin or stained with Coomassie blue as the protein loading control for Western blot analysis.

To explain the finding of DDB2 knockdown causing reduced growth and a prolonged doubling time of MCF-7 cells, cell cycle analysis was performed by flow cytometry, after PI staining. Wt, siRNA control and DDB2-deficient MCF-7 cells were synchronized by serum starvation for 48 h and then induced to re-enter the cell cycle by the addition of serum. The cell lines were exposed to added serum over 0, 3, 12 or 18 h. The cell cycle distribution of the cell lines was determined by quantifying DNA content, after PI staining (Figure 5). After PI staining, serum depletion for 48 h was shown to result in G1/G0 and G2/M arrests of at least 98% in control (Wt and siRNA control) and DDB2-deficient MCF-7 cells (cl.2 and cl.3). At 3 h after serum addition, no change in cell cycle distribution was observed for the DDB2-deficient MCF-7 cell lines, while the S-phase fraction increased in the Wt and siRNA control cells (11 and 9%, respectively). With the time release from serum addition (12 and 18 h), we observed that both DDB2-deficient MCF-7 cell lines re-entered the cell cycle, with a loss of G1 cells and the appearance of a high S-phase fraction, but no G2 cell presence. The high peak corresponding to the S-phase fraction shifted slightly between 12 and 18 h after serum addition. However, the control MCF-7 cells were distributed in all phases of the cell cycle, with a lower S-phase fraction and a significant peak corresponding to about 19% and 14% G2 cells after 12 and 18 h addition of serum, respectively, compared to the DDB2-deficient MCF-7 cell lines. These results suggest that the DDB2 deficient MCF-7 cells advanced in the cell cycle more slowly than the control cells. These results were confirmed by 5 BrdU incorporation into cells growing synchronously after serum starvation. The cell fractions in G1/S and S-phases were estimated by flow cytometry analysis (Figure 6). A lack of labeling index (LI) corresponding to a lack of 5 BrdU incorporation was observed, indicating no cells were in the S-phase after 48 h of serum starvation. An alteration of cell cycle progression was observed in the DDB2 deficient MCF-7 cells 3 h after the addition of serum. Similar to the finding by cell cycle analysis after PI staining, no 5 BrdU incorporation was quantified for both DDB2-deficient MCF-7 cell lines, whereas the LI for the Wt and siRNA control MCF-7 cells (6.6% and 9.3%, respectively) revealed important S-phase fractions in these lines. Then, % 5 BrdU-positive cells corresponding to the LI for DDB2 deficient cells was strongly increased and was similar to the control MCF-7 cells at 12 and 18 h after release from serum depletion. Compared to that of the control cells, this LI indicated an important pool of DDB2-deficient cells which started to re-enter the cell cycle and which corresponded to an essentially G1/S subpopulation. The DDB2-deficient MCF-7 cell clones 2 and 3 showed S-phase fractions respectively 6.8- and 4.2-fold less than that of the control MCF-7 cells, at 12 and 18 h after release from serum depletion. In addition, no G2 fraction was detected for both DDB2-deficient MCF-7 cell clones. These results demonstrate that DDB2 knockdown led to a delayed G1/S transition phase entry and a slowed MCF-7 cell progression through the S phase. These results were confirmed by an investigation of the PCNA protein level. Regardless of the time from release of serum depletion, the PCNA protein level was greatly reduced in both DDB2-deficient MCF-7 cell lines, compared to that of tubulin, used as a loading control (Figure 7).

## Discussion

In addition to its role in DNA repair, DDB2 could play an important role in cell cycle regulation in normal cells, when associated with proteins regulating the cell cycle [7], [8]. Previous studies showed that mutations in the DDB2 gene, which lead to a deficiency in the NER system, increased susceptibility to develop cancer [21] and a DDB2 deficiency promoted spontaneous malignant tumors in mice [17], [22]. Taken together, these results provided evidence that DDB2 could play a role as a tumor suppressor in normal cells. However, strong evidence for the functional interaction between DDB2 and E2F1, a proliferative marker in many cancers led us to speculate that DDB2 could be involved also in cancer cell growth [8], [20].

The present study reports for the first time that DDB2 is overexpressed in ER-positive breast cancer cell lines, compared to the constitutive DDB1 expression. As described already in other cell types, DDB2 was localized strictly in the nucleus, as demonstrated by the presence of several nuclear localization signals in its amino acid sequence [23], whereas DDB1 protein, which is expressed constitutively in cells, was found to be localized also in the cytoplasm and nucleus [24]. DDB2 was detected as two bands by Western blot analysis in MCF-7 and T47D cells, but only the one with the higher molecular weight was found in the nuclear extract: the second was suspected to be non-specific and the result of cross-reaction with anti-DDB2. The basal DDB2 level was very low or undetectable in ER-negative breast cancer cells and in the nontumorigenic epithelial mammary HMEC cell line. In addition, a correlation between DDB2 expression and ER status was observed also in breast tumor samples from patients. Taken together, we postulate that a high DDB2 content is associated with the oestrogen receptor (ER) status in breast cancer.

Expression of the DDB2 gene has been detected also in some tumor cell lines such as colon carcinoma cell lines and HeLa cells [11], [25]. It is known that the DDB2 gene is expressed at a low baseline level in many human normal cells and is induced following UV irradiation to participate in the repair of DNA lesions [15], [23]. Regulation of DDB2 expression is not well known. A recent study identified Sp1, NF1 and E2F response elements in the promoter of the DDB2 gene, and showed that mutations of these response elements reduced strongly the basal transcription of the DDB2 gene [24], [26]. In addition, it has been found that p53 and BRCA1 were able to activate the DDB2 gene [9], [10]. In the present study, we observed that the DDB2 gene was upregulated in ER-positive breast cancer cells, compared to the very low expression of DDB2 in the nontumorigenic epithelial mammary HMEC cell line. The mechanism by which DDB2 expression is dysregulated in ER-breast cancer cells is not known. Moreover, the molecular mechanism involved in the loss of DDB2 gene expression in ER-negative breast cancer cells will need to be defined in the future. One hypothesis would suggest the involvement of BRCA1. The ER-positive breast cancer cells, such as MCF-7 and T47D cells, express BRCA1, whereas the ER-negative tumor cells, such as SKBR3 and MDA-MB231 cells are BRCA1 negative [27]. Involvement of ER and other transcription factors or other molecular mechanisms are not excluded and future investigations will need to elucidate the regulation of DDB2 expression during breast tumor progression.

The surprising evidence that the high DDB2 content correlated with the high proliferation rate of MCF-7 and T47D cells compared to MDA-MB231 and SKBR3 cells, along with a number of studies reporting a role of DDB2 in the cell cycle regulation of normal cells, led us to investigate the role of this protein in tumor growth. The result of the inhibition of DDB2 expression, through the strategy of small interfering RNA (siRNA), gave a significant reduction of the growth rate and clonogenicity of the MCF-7 cells and an increased cell doubling time. Inversely, introduction of the DDB2 gene into MDA-MB231 cells increased their growth rate and clonogenicity and decreased their cell doubling time. We demonstrated that the increase in the doubling time of DDB2-deficient MCF-7 cells resulted in a slowing of their entry into G1/S transition and of their progression through the S phase of cell cycle. Moreover, no G2 fraction was detected 18h after release from serum depletion for both DDB2-deficient MCF-7 cell clones compared to control cells. These data suggest that DDB2 may play a role as an activator of breast tumor cell proliferation at the G1/S transition and during the S-phase progression of the cell cycle. The involvement of DDB2 at this checkpoint of the cell cycle correlates with the fact that it is well expressed in dividing and undamaged normal cells in the mid-G1 phase and peaks at the G1/S boundary, before dropping significantly in the S phase [12].

A strong decrease in the expression of DHFR, cyclin E and PCNA genes, which are required for DNA synthesis and more generally for proliferation, was observed in DDB2-deficient MCF-7 cells. These results suggest that DDB2 deficiency, by promoting a down-regulation of the replication genes during tumor cell growth, leads to the slowed entry into the G1/S transition and the S phase of the cell cycle. Expression of these genes is controlled by E2F1, a transcription factor which stimulates cell cycle progression at G1 to S phase [28]. This role is well documented in tumor cells, and particularly in breast cancer cells where E2F1 and its E2F1-target genes, including DHFR, PCNA and cyclin E, are overexpressed [20], [29]. Also, one mechanism by which DDB2 plays a role in tumor cell proliferation could be related to its interaction with E2F1. Indeed, in association with DDB1, DDB2 has a functional interaction with E2F1, leading to the stimulation of the E2F1-target gene expression [8]. However, no significant correlation between E2F1-target genes and DDB2 was observed in our breast tumor samples (see Supplemental Figure S3). This preliminary clinical study was probably limited by the small number of samples. Also, further investigation with larger breast tumor samples will be necessary.

The present study provides new insights showing that DDB2 plays a novel biological function. DDB2 functions as a tumor suppressor in normal cells, at least in part by regulating cell proliferation and controlling p53-mediated apoptosis. Deletion of DDB2 in normal cells promotes spontaneous tumor growth in the absense of UV- or carcinogen-induced DNA lesions, probably due to an accumulation of unrepaired DNA lesions, leading to cell transformation [17]. In this study, DDB2 exhibited in vitro oncogenic properties in ER-positive breast cancer cells, through the stimulation of cell proliferation. These results suggest that the overexpression of DDB2 imparts a growth advantage for ER-positive breast cancer cells. We can consider that DDB2 exhibits ying and yang activity, as does E2F1. Under normal circumstances, E2F1 plays a role in stimulating growth through its regulation of the expression of genes required for cell cycle progression in breast cancer cells [20], [30]. On the other hand, E2F1 can also play a role in cell cycle arrest and apoptosis, in response to DNA damage [31].

Our results describe an association between DDB2 and ER status in breast cancer as well as in preclinical models and in clinical specimens. It is known that the absence of ER in breast tumor cells is associated with a poor prognosis and an aggressive phenotype. The ER-negative breast cancer cell models used in this study, such as MDA-MB231 and SKBR3 cells are aggressive and highly metastatic estrogen-independent breast cancer cells because of high invasive ability, despite a slow growth rate. The increase in MDA-MB231 cell proliferation, which was observed after introduction of the DDB2 gene, suggests that DDB2 protein is a candidate oncogene when it is overexpressed, whatever the breast cancer cell phenotype. Also, the down-regulation of the DDB2 gene observed in these cells may reflect tumor progression to a metastatic phenotype. It is not excluded that the decrease in DDB2 expression is associated with a decrease in the NER system and an accumulation of unrepaired DNA damage, leading to an invasive cancer phenotype [32]. In addition to causing impairment in the DNA repair function, the loss of DDB2 gene expression, leading to a slowed growth of aggressive breast cancer cells, may be proposed as an important step in mammary carcinogenesis in ER-negative cells. Also, DDB2 may represent an important clinical interest as a prognostic/predictive marker of breast cancer progression towards an aggressive phenotype. A larger clinical investigation is needed to support this hypothesis.

The present study provides for the first time strong evidence that DDB2 is a candidate for oncogene action in breast cancer. It may contribute to a better understanding of breast tumor progression and to provide a valuable tool in the clinical investigation of breast cancer. This conclusion is based on several lines of evidence that DDB2 is highly expressed in the human ER-positive breast tumor samples and in the cell lines we examined, compared to ER-negative status, and plays a significant role as an activator of growth, favouring G1/S transition entry of ER-positive breast cancer cells during their cell cycle. These findings support the assessment of DDB2 status as a prognostic factor for tumor progression and chemosensitivity in breast cancer and, possibly, other cancers. Elevated levels of DDB2, through its ability to activate cancer cell growth, may influence the effectiveness of anticancer drugs. Also, the molecular mechanism by which the factors and the signaling pathway influencing DDB2 expression in ER-positive breast cancer cells will need to be defined.

## Materials and Methods

### Cell Lines

Human ER-expressing (MCF-7 and T47D), ER-negative (MDA-MB231 and SKBR3) breast cancer cell lines and normal human mammary epithelial cells (HMEC) from Clonetics (Cambrex) were cultured as described previously [33].

### Tumor material

Sixteen anonymous (eight estrogen receptor positive, eight negative), 20-30 mg breast tumor samples were from surgical pieces of infiltrating breast carcinomas from patients in intend to make diagnosis before an appropriate treatment. According to the French bioethical law of august 6, 2004 (number 2004-800), which has been then completed by the law of august 10, 2007 (number 2007-1220, NOR: ESRR 0757 103D), samples used for this retrospective study did not required written consent from patients. These samples were kept in liquid nitrogen frozen specimens and then were certified as tumor material by morphological characterization by a senior pathologist. Estrogen receptor expression was analyzed at diagnosis using routine immunohistochemistry with 6F11 monoclonal antibody determination kit (Novocastra) and revealed using I-View DAB kit (Ventana). Estrogen receptor expression was considered as positive when more than 10% of the nuclei were stained. Semi-quantitative expression values were calculated as % labeled nuclei×staining intensity (0–3).

### Reverse Transcription-Polymerase Chain Reaction (RT-PCR) Analysis

Total RNA from breast cancer cell lines and from 16 frozen human tissues samples was isolated with Trizol® (Invitrogen). RNA quality from human breast cancers was controlled using RNA nanoLab Chip® (2100 Bioanalyzer, Agilent Technologies) and used for RT-PCR. One microgram of total RNA was reverse-transcribed for 50 min at 42°C in 20 µl of PCR buffer with 2.5 mM dNTPs, 5 µM random hexamer primers, 1.5 mM MgCl_2_ and 200 units SuperScript II reverse transcriptase. The primers used (Invitrogen) were selected from published nucleotide sequences in the open reading frames of the human genes encoding DDB1 and DDB2 [34]. The primer sequences used were as follows: DDB1 forward, 5′-GACCTGCCCTACGACTAC-3′; DDB1 reverse, 5′-GACCACCACCATTGAACTTC-3′; DDB2 forward, 5′-GCGACGAAGGCCGTGTGCGTGC-3′; DDB2 reverse, 5′-ACTTTCTTCATTTCCACCTTTGCC-3′; dihydrofolate reductase (DHFR) forward, 5′-TGGCTCACACCTGTAATCC-3′; DHFR reverse, 5′- TAATTCTTCCATCTCAGCTTCC-3′; Proliferating Cell Nuclear Antigen (PCNA) forward, 5′-TGCGGCCGGGGTTCAGGAGTCA-3′; PCNA reverse, 5′-CAGGCAGGCGGGAAGGAGGAAAGT-3′; cyclin E forward, 5′-TATTGCAGCCAAACTTGAGG-3′; cyclin E reverse, 5′-TTAGATATGCAACCTGCATGTATAC-3′; β-actin forward, 5′-GGCTCCGGCATGTGCAAGG-3′; β-actin reverse, 5′-AGATTTTCTCCATGTCGTCC-3′. Each primer was added at a final concentration of 0.5 µM to 50 µl reaction mixture in PCR buffer, containing 1 µl cDNA, 0.25 mM of each dNTP, 1.5 mM MgCl_2_, and 2.5 units Taq polymerase. An initial denaturation was carried out for 2 min at 94°C and 30 cycles were performed with the following PCR program : denaturing 94°C-45 s, annealing 50°C for DDB1 and DBB2 or 46°C for DHFR or 56°C for PCNA or 45°C for cyclin E and β-actin-45 s, elongation 72°C-45 s. This program was completed with a final extension of 5 min at 72°C. Preliminary assays have shown that the 30 cycle amplification was in the exponential phase. Ethidium bromide-stained bands were visualized by UV transillumination and the fluorescence intensity was quantified using a Gel Doc 2000 system (Biorad). The data from PCR reactions were expressed as the relative DDB2 or DDB1 mRNA level, corresponding to the ratio of the quantified fluorescence intensity of DDB1 or DDB2 band/β-actin band from three independent experiments±standard deviations (SD). DNA fragments (with expected sizes of 300 bp for DDB1 and DDB2, 172 bp for DHFR, 348 bp for PCNA, 198 bp for cyclin E and 220 bp for β-actin) were purified with the Prep A gene DNA purification matrix kit (Biorad), and their sequences were determined according to the dideoxy chain-termination method [35] and were found identical to those published previously.

### Preparation of Total, Nuclear and Cytoplasmic Extracts

Human breast cancer cell lines were harvested and lysed in a 10 mM Tris/HCl buffer, pH 7.4, containing 5 mM EDTA, 1% Triton X100 and protease inhibitor cocktail, at 4°C for 20 min. After centrifugation at 17,000 g for 20 min at 4°C, the supernatant was collected as total protein extract. Nuclear and cytoplasmic extracts were prepared as described previously [36]. The cells were rinsed twice with PBS and were scraped with a rubber policeman in PBS. After a brief centrifugation at 100 g for 5 min at 4°C, the cells were resuspended in 10 mM Hepes, pH 7.9, containing 10 mM KCl, 0.1 mM EDTA and EGTA, 1 mM dithiotreitol, and 0.5 mM phenylmethylsulfonyl fluoride and incubated for 15 min on ice. The cells were gently lysed by the addition of 0.6% (v/v) Nonidet P-40 and centrifuged at 200 g and 4°C for 5 min. The supernatant was collected as the cytoplasmic extract. Pelleted nuclei were resuspended and lysed in 20 mM Hepes, pH 7.9, containing 400 mM NaCl, 1 mM EDTA and EGTA, 1 mM dithiotreitol, 0.5 mM phenylmethylsulfonyl fluoride and 0.25% (v/v) Nonidet P-40. After centrifugation at 12,000 g for 10 min and at 4°C, the supernatant was collected as nuclear extract. Protein concentrations were determined in the total, nuclear and cytoplasmic extracts according to Lowry *et al.*, [37], using bovine serum albumin as a standard (Biorad).

### Western Blot Analysis

Total proteins (50 µg), nuclear proteins (20 µg) and cytoplasmic proteins (30 µg) were run on SDS-polyacrylamide gels (12%), according to Laemmli [38], and transferred onto a PVDF membrane as described previously [39]. Immunoblot analysis was then carried out using specific polyclonal anti-DDB1, anti-DDB2, anti-Histone H1 (Santa Cruz Biotechnology) and anti-catalase [40] at the optimized dilutions. Bands were detected using an anti-IgG polyclonal antibody conjugated to peroxidase (Sigma), after exposition to a chemiluminescent substrate. Band intensities were quantified by densitometry with a Gel Doc 2000 system (Biorad). The results from Western blots were expressed as relative densitometric units from three independent experiments±SD. Equal loading of protein in all experiments was confirmed by Coomassie blue staining of blots.

### In Situ Immunofluorescence

The cells were seeded at 1×10^4^cells/well in a 4 chamber slide (NUNC Lab-Tek II, Euromedex) and incubated at 37°C for 5 days before confluence. The cells were rinsed twice with PBS and fixed in 3% formaldehyde in PBS for 10 min and permeabilized in methanol for 20 min at 4°C. After blocking with 0.1% fish gelatin/0.8% bovine serum albumin/0.002% Tween-80, the cells were then exposed to the primary polyclonal antibodies anti-DDB1, anti-DDB2 and anti-proliferating cell nuclear antigen (PCNA) (Santa Cruz Biotechnology), diluted at 1∶100, for 30 min at 37°C. After two washes in PBS, the cells were incubated with FITC-conjugated bovine anti-rabbit immunoglobulins, diluted at 1∶100 (Santa Cruz Biotechnology), in PBS for 20 min at 37°C. A negative control was performed without the primary antibody. The cells were then mounted in anti-fading medium (Citifluor, Link Analytical). Images of cellular immunofluorescence were acquired using an epifluorescence microscope Eclipse 80i with 40X objective (488 nm excitation and 518 emission) and captured with a coupled digital camera (Nikon) (See Supplemental data).

### DDB2 Expression Vector and Transfection

The full-length human DDB2 cDNA containing the entire open reading frame (1300 bp) was isolated from MCF-7 cells by RT-PCR using the Hi-fidelity Extensor PCR kit (AB Gene), and the forward (5′-GGACTGGGTACCACACGGAGGACGCGATGGCTC-3′) and the reverse primers (5′-CTGAGCTCTAGATCACTTCCGTGTCCTGGCTTCC-3′), with *Kpn I* and *Xba I* ends, respectively, according to the manufacturer's instructions. The resulting DDB2 cDNA was inserted between the *KpnI* and *XbaI* sites into a pcDNA3.1(+) mammalian expression vector (Invitrogen), driven by a cytomegalovirus promoter. The complete sequence of the cDNA was verified by DNA sequence analysis. The DDB2 cDNA was also subcloned into a pEF1/Myc-HisB vector (Invitrogen) between the *KpnI* and *XbaI* sites, to produce a Myc-polyhistidine-tagged DDB2 protein (See Supplemental data). The expression vectors included a Neo resistance gene driven by the SV40 promoter for clone selection. The size of the recombinant protein was verified by using the wheat germ lysate transcription-translation TNT kit (Promega) according to the manufacturer's instructions. Four µg of pcDNA3(+) or pEF1/Myc-HisB plasmid containing either DDB2 cDNA or no insert were used for stable transfection of MDA-MB231 or COS-7 cells, with TransPEI reagent (Eurogentec), according to the manufacturer's instructions. The clones were selected with 800 µg/ml of G418 for 4 weeks. Single colonies were isolated and then screened for levels of the expression of DDB2 protein by Western blot analysis. Five days before these experiments, the cells were placed into complete medium without G418 supplement.

### DDB2-siRNA Vector and Transfection

SiRNA oligonucleotides were obtained from Eurogentec in a purified and annealed duplex form. The sequences targeting the human DDB2 gene are: target 1 for DDB2, 5′-AGAGCGAGAUCCGAGUUUAA-3′ (sense) and 5′-UAAACUCGGAUCUCGCUCUU-3′ (antisense); target 2 for DDB2, 5′-UCAGUUCGCUUAAUGAAUUU-3′ (sense) and 5′-AAUUCAUUAAGCGAACUGAA-3′ (antisense); target 3 for DDB2, 5′-UCACUGGGCUGAAGUUUAA-3′ (sense) and 5′-UUAAACUUCAGCCCAGUGAA-3′ (antisense). Scrambled siRNA with the following sequence: 5′-UUAAACUUCAGCCCAGUGA-3′ (sense) and 5′-CAGUAAACGCCGUCUUAUA-3′ (antisense) was used as the control. SiRNA transfection experiments were carried out using jetSi-ENDO transfection reagent with 100 nM siRNA, according to the manufacturer's instructions (Eurogentec). Twenty-four hours following siRNA transfection, the cells were used to analyze the expression of DDB2 protein (see Supplemental data). Double strand DNA oligonucleotide encoding the effective siRNA in the knockdown of DDB2 was synthesized with a loop sequence TTCAAGAGA and a RNA pol III terminator sequence consisting of a 6 poly T. This double strand DNA oligonucleotide was cloned into the RNAi-ready pSIREN vector (BD Biosciences Clontech) between the *BamHI* and *EcoRI* restriction sites with the U6 promoter. This vector contains a puromycin resistance gene for the selection of stable transfectants. A unique *Xba I* restriction site was placed downstream of the terminator sequence for restriction digest analysis to confirm the presence of the cloned insert. Four µg of pSIREN/U6/DDB2-siRNA vector or pSIREN/U6 empty vector were used for stable transfection of MCF-7 cells with TransPEI transfection reagent, according to the manufacturer's instructions (Eurogentec). The MCF-7 clones were selected with 0.5 µg/ml of puromycin for 3 weeks. Single colonies were isolated and then screened for levels of the expression of DDB2 protein by RT-PCR and Western blot analyses. Five days before the experiments, the cells were placed into complete medium without puromycin supplement.

### Cell Growth

Cells (1×10^4^) were plated in 24-well dishes. The cell growth rate was determined by counting the number of cells with a hemocytometer as a function of time. Cell population doubling time (Td) was calculated from the growth rate during the exponential growth using the following formula: *Td = 0.693t/ln(N_t_/N_0_)*, where *t* is time in days, *N_t_* is the cell number at time, and *N_0_* is the cell number at the initial time. The data from cell growth were expressed as means±SD from at least three independent experiments, each being performed in triplicate.

### Colony Formation

Cells (5×10^2^) were plated in 100-mm culture dishes and incubated for 12 days to allow colony formation. The colonies were then fixed in ethanol, stained with 0.1% crystal violet and scored when they contained more than 50 cells. Results were expressed as follows: colony formation (%) = (colonies formed/cells seeded)×100%. The data from colony formation were expressed as means±SD from at least three independent experiments, each being performed in triplicate.

### Flow Cytometry Analysis

Cells (2×10^4^/ml) were plated in 75 cm^2^ culture dishes and grown in complete RPMI 1640 culture medium. After a 3-day culture, the cells were washed three times with PBS and then synchronized by serum starvation for 48h. The cells were then induced to re-enter the cell cycle by the addition of serum for 0, 3, 12 or 18h and were harvested by trypsinization. The pellet of cells was resuspended in 0.1% sodium citrate, 0.1% Triton X100 and 50 µg/ml propidium iodide (PI), and then stored for 24h at 4°C. After centrifugation at 300 g for 5 min, the cells were resuspended in PBS containing 50 µg/ml of RNAse, and the DNA content was determined by Fluorescence-activated cell sorting (FACS) analysis using an Orthocyte flow cytometer (Ortho Diagnostic Systems). To aid in the determination of the ability of the serum-starved cells to re-enter the S phase of the cell cycle, 100 µM of 5 Bromodeoxyuridine (BrdU) were added to the culture medium for 20 min at the end of each incubation with serum (0, 3, 12 or 18h). Cells suspensions were prepared as described previously [41], using the FITC-coupled anti-BrdU monoclonal antibody provided by Dako and were then analyzed by FACS. The data were analyzed using Cell Quest sofware (BD Biosciences Clontech). The Labeling Index (LI) corresponded to the percentage of BrdU-positive cells. The G1/S subpopulation, corresponding to BrdU-positive cells containing G1 DNA and S fractions, was calculated from the LI and expressed as the percentage of 5 BrdU-positive cells.

### Statistical Analysis

Evaluation of statistical significance for data from RT-PCR, Western blots, cell growth and colony formation was assessed using analyses of variance (ANOVA) and the Fisher protected least significant difference test. Statistical significance was indicated as *P<0.05. Statistical analysis for breast cancer samples from patients was performed using the Mann Whitney test. Correlation between different gene expressions was performed with Pearson correlation coefficient method. Differences were considered to be statistically significant at a value of *P*<0.05.

## Supporting Information

Figure S1 Localization of DDB1 and DDB2 in MCF-7 cells by immunocytochemistry. (A) DDB1 and DDB2 were detected by indirect immunofluorescence using the respective polyclonal antibodies. PCNA corresponding to the positive control was also detected by a specific polyclonal antibody. Negative control was performed without the primary antibody. (B) The presence of DDB1 and DDB2 were detected by Western blotting in total (50 µg), nuclear (20 µg) and cytoplasmic proteins (30 µg), using specific polyclonal antibodies. Positive controls corresponding to the cytoplasmic catalase and the nuclear histone H1 were detected by Western blotting with the respective polyclonal antibodies.
(0.11 MB TIF)

Figure S2 Identification of DDB2-specific siRNA suppressing DDB2 protein level in MCF-7 cells and Poly His tagged DDB2-overexpressing COS-7 cells. (A) MCF-7 cells were transfected with 100 nM of three different DDB2-specific siRNA for 24h. Suppression of DDB2 protein level was assessed by Western blot analysis using equal amounts of protein (50 µg) and the anti-DDB2 polyclonal antibody. Results were compared to the non-transfected cells (-) and to the scrambled siRNA-transfected cells (C). (B) COS-7 cells were stably transfected either with empty vector-transfected cells (Neo) or with His-Myc tagged DDB2 expression vector. Myc-His tagged DDB2 overexpression was verified by Western blot analysis and is indicated by an arrow. (C) Myc-His tagged DDB2 overexpressing-COS-7 cells were transfected with 100 nM of the three different DDB2-specific siRNA for 24h. Suppression of Myc-His tagged DDB2 protein level was assessed by Western blot analysis using equal amounts of protein (50 µg) and results were compared to those from Myc-His tagged DDB2 overexpressing-COS-7 cells without siRNA (-) or transfected with the scrambled siRNA (C).
(0.06 MB TIF)

Figure S3 PCNA, cyclin E and DHFR expression in human breast tumors from patients. Total RNA was extracted from eight ER-positive and eight ER-negative breast cancer samples, then subjected to semiquantitative RT-PCR analysis. (A) The relative levels of PCNA, cyclin E and DHFR mRNAs were normalized to those of β-actin mRNA. Statistically significant differences between ER-positive and ER-negative samples are indicated as *P*<0.05. The mean values are indicated by a bar in graph for each group of tumors and PCNA, cyclin E or DHFR mRNA levels. (B) Correlation between relative PCNA, cyclin E or DHFR and DDB2 mRNA levels was performed with Pearson correlation coefficient method. Differences were considered to be statistically significant at a value of *P*<0.05.
(0.04 MB TIF)
