# Supplementary figures and images for: Damaged DNA Binding Protein 2 Plays a Role in Breast Cancer Cell Growth
Source: PLoS One. 2008 Apr 23;3(4):e2002. doi: 10.1371/journal.pone.0002002 (PMC2291195; doi:10.1371/journal.pone.0002002)

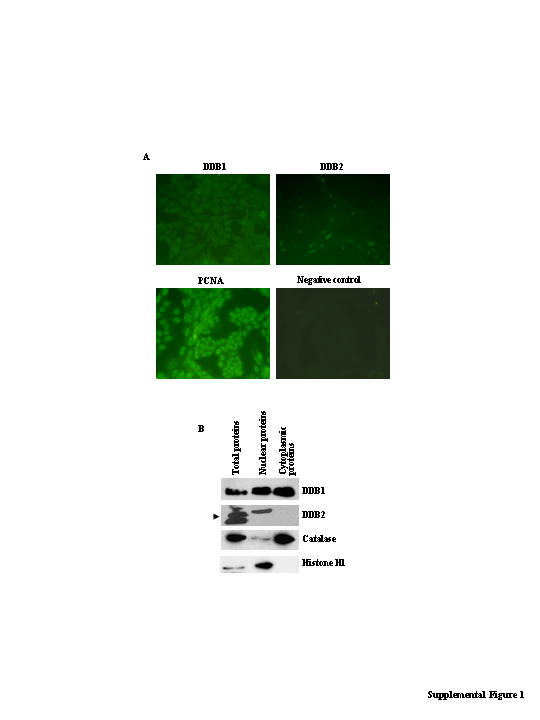

Supplement: Figure S1 — Localization of DDB1 and DDB2 in MCF-7 cells by immunocytochemistry. (A) DDB1 and DDB2 were detected by indirect immunofluorescence using the respective polyclonal antibodies. PCNA corresponding to the positive control was also detected by a specific polyclonal antibody. Negative control was performed without the primary antibody. (B) The presence of DDB1 and DDB2 were detected by Western blotting in total (50 µg), nuclear (20 µg) and cytoplasmic proteins (30 µg), using specific polyclonal antibodies. Positive controls corresponding to the cytoplasmic catalase and the nuclear histone H1 were detected by Western blotting with the respective polyclonal antibodies. (0.11 MB TIF) [file pone.0002002.s001.tif]

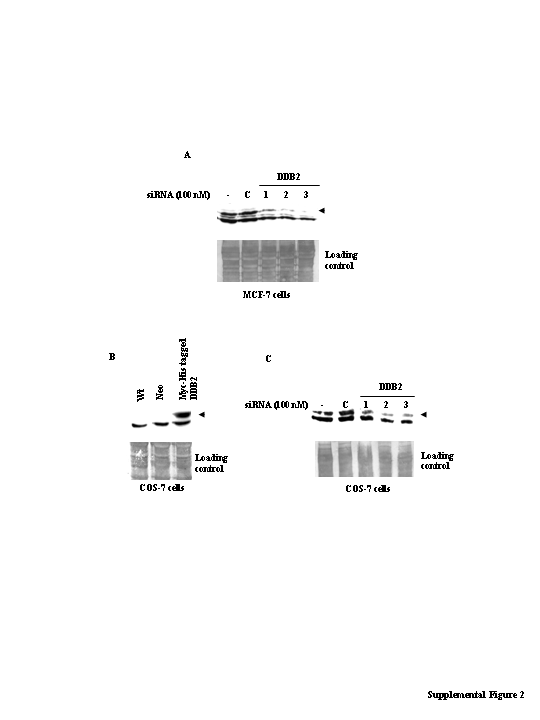

Supplement: Figure S2 — Identification of DDB2-specific siRNA suppressing DDB2 protein level in MCF-7 cells and Poly His tagged DDB2-overexpressing COS-7 cells. (A) MCF-7 cells were transfected with 100 nM of three different DDB2-specific siRNA for 24h. Suppression of DDB2 protein level was assessed by Western blot analysis using equal amounts of protein (50 µg) and the anti-DDB2 polyclonal antibody. Results were compared to the non-transfected cells (-) and to the scrambled siRNA-transfected cells (C). (B) COS-7 cells were stably transfected either with empty vector-transfected cells (Neo) or with His-Myc tagged DDB2 expression vector. Myc-His tagged DDB2 overexpression was verified by Western blot analysis and is indicated by an arrow. (C) Myc-His tagged DDB2 overexpressing-COS-7 cells were transfected with 100 nM of the three different DDB2-specific siRNA for 24h. Suppression of Myc-His tagged DDB2 protein level was assessed by Western blot analysis using equal amounts of protein (50 µg) and results were compared to those from Myc-His tagged DDB2 overexpressing-COS-7 cells without siRNA (-) or transfected with the scrambled siRNA (C). (0.06 MB TIF) [file pone.0002002.s002.tif]

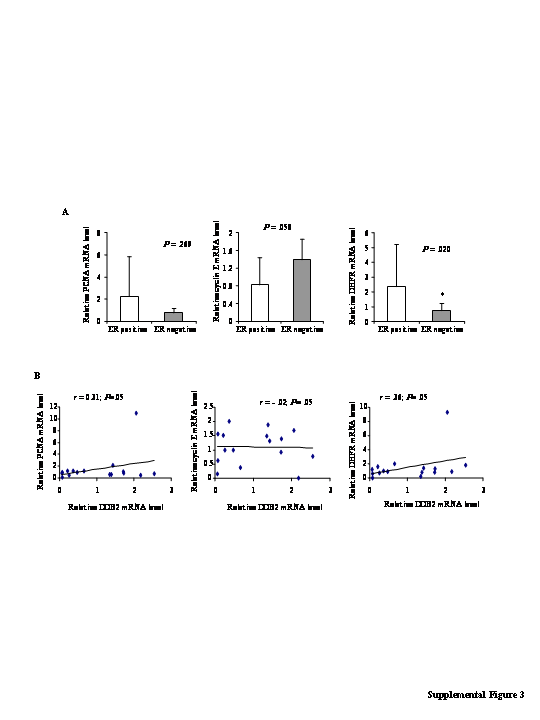

Supplement: Figure S3 — PCNA, cyclin E and DHFR expression in human breast tumors from patients. Total RNA was extracted from eight ER-positive and eight ER-negative breast cancer samples, then subjected to semiquantitative RT-PCR analysis. (A) The relative levels of PCNA, cyclin E and DHFR mRNAs were normalized to those of β-actin mRNA. Statistically significant differences between ER-positive and ER-negative samples are indicated as P<0.05. The mean values are indicated by a bar in graph for each group of tumors and PCNA, cyclin E or DHFR mRNA levels. (B) Correlation between relative PCNA, cyclin E or DHFR and DDB2 mRNA levels was performed with Pearson correlation coefficient method. Differences were considered to be statistically significant at a value of P<0.05. (0.04 MB TIF) [file pone.0002002.s003.tif]
